# Medical Schools—Medical Politics

**Published:** 1857-07

**Authors:** 


*Medical Schools—Medical Politics.*

Our city has been full of rumors for some time past, in reference to a proposed "consolidation" between the Medical College of Ohio and the Miami Medical College. After considerable diplomacy, an arrangement has been consummated whereby the latter school ceases to exist, and four members of its faculty. Drs. Judkins, Comegys, Foote and Mendenhall go into the former; Drs. Armor, Marshall, Warder and Tate withdrawing. We understand the faculty of the Ohio School, as thus constituted, together with the board of trustees, heartily unite in certain general plans for the future, that, if fully carried out, promise to increase the facilities and usefulness of the school. A fuller *Hospital Service* is contemplated. The students and alumni of the Miami School are to be placed on an equal footing with a corresponding attendance or position at the Ohio School, graduates of the Miami School being entitled to a diploma from the Ohio Medical College, if desired. At present we have no comments to make upon this new arrangement. We wish it success, but are not committed for it or against it. To our patrons we have to say, however, this will not make any change whatever, at least at present, in the Observer. This journal is private property, and is not comprehended in any of these "consolidated" enterprises.—*Med. Observer.*

